# Improving Handover Between Psychiatric and Acute Wards

**DOI:** 10.1192/bjo.2024.585

**Published:** 2024-08-01

**Authors:** Hilary James-Thomson

**Affiliations:** Forth Valley Royal Hospital, Falkirk, United Kingdom

## Abstract

**Aims:**

1. To develop an understanding of transfers in both directions between Psychiatry and the other Acute wards within FVRH.
2. To improve compliance with National standards of handover as laid out by NICE and GMC.

**Methods:**

Data was taken from a combination of the care partner, trakcare, and HepMA systems looking at the quality, content and professionals involved in handover. This was done for all transfers in and out of 2 old age psychiatry wards over a 2 week period. The auditing of transfers into MHU and transfers out of MHU initially started as 2 separate projects with staggered data collection; these were combined after baseline data was collected, and the intervention and re-audit phases treated the two as a single project. Data was compared with the standards collated from GMC and NICE guidance.

**Results:**

Transfer handovers in neither direction met audit standards at baseline assessment.

Interventions focusing on behavioural change in the mental health unit did achieve behavioural change but failed to solve issues with handover between departments.

It is worth noting that there was significant delay in some transfers out of mental health being escalated, considering the reduced facilities in mental health wards versus acute wards.

**Conclusion:**

Transfer handover between Psychiatry and Acute Wards is a multi-system issue and as such will require a multi-system approach to achieve meaningful change. New local guidance for handover between mental health and acute wards is being drafted in response to the findings of this audit.

